# Comparative *in vitro *activity of Meropenem, Imipenem and Piperacillin/tazobactam against 1071 clinical isolates using 2 different methods: a French multicentre study

**DOI:** 10.1186/1471-2334-10-72

**Published:** 2010-03-18

**Authors:** Marie-Laure Joly-Guillou, Marie Kempf, Jean-Didier Cavallo, Monique Chomarat, Luc Dubreuil, Jeanne Maugein, Claudette Muller-Serieys, Micheline Roussel-Delvallez

**Affiliations:** 1 Bacteriology department, Universitary Hospital, (Larrey St), Angers (49000), France; 2 Bacteriology department, Begin Military Hospital, (Paris Ave), St Mandé, (94163), France; 3 Bacteriology department, Universitary Hospital Lyon-Sud, Pierre Bénite, (69310), France; 4 Faculty of Pharmacy, (Dr Laguesse St), Lille, (59006), France; 5 Bacteriology department, Haut Leveque Hospital, (Magellan Ave), Pessac, (33604), France; 6 Bacteriology department, Universitary Hospital Bichat Claude Bernard, AP-HP, Paris, (75018), France; 7 Bacteriology department, Universitary Hospital Calmette, (J. Leclercq Bd), (59037), Lille, France

## Abstract

**Background:**

Meropenem is a carbapenem that has an excellent activity against many gram-positive and gram-negative aerobic, facultative, and anaerobic bacteria. The major objective of the present study was to assess the *in vitro *activity of meropenem compared to imipenem and piperacillin/tazobactam, against 1071 non-repetitive isolates collected from patients with bacteremia (55%), pneumonia (29%), peritonitis (12%) and wound infections (3%), in 15 French hospitals in 2006. The secondary aim of the study was to compare the results of routinely testings and those obtained by a referent laboratory.

**Method:**

Susceptibility testing and Minimum Inhibitory Concentrations (MICs) of meropenem, imipenem and piperacillin/tazobactam were determined locally by Etest method. Susceptibility to meropenem was confirmed at a central laboratory by disc diffusion method and MICs determined by agar dilution method for meropenem, imipenem and piperacillin/tazobactam.

**Results:**

Cumulative susceptibility rates against *Escherichia coli *were, meropenem and imipenem: 100% and piperacillin/tazobactam: 90%. Against other *Enterobacteriaceae*, the rates were meropenem: 99%, imipenem: 98% and piperacillin/tazobactam: 90%. All *Staphylococci*, *Streptococci *and anaerobes were susceptible to the three antibiotics. Against non fermeters, meropenem was active on 84-94% of the strains, imipenem on 84-98% of the strains and piperacillin/tazobactam on 90-100% of the strains.

**Conclusions:**

Compared to imipenem, meropenem displays lower MICs against *Enterobacteriaceae*, *Escherichia coli *and *Pseudomonas aeruginosa*. Except for non fermenters, MICs90 of carbapenems were <4 mg/L. Piperacillin/tazobactam was less active against *Enterobacteriaceae *and *Acinetobacter *but not *P. aeruginosa*. Some discrepancies were noted between MICs determined by Etest accross centres and MICs determined by agar dilution method at the central laboratory. Discrepancies were more common for imipenem testing and more frequently related to a few centres. Overall MICs determined by Etest were in general higher (0.5 log to 1 log fold) than MICs by agar dilution.

## Background

Antimicrobial susceptibility surveillance programmes represent one of the main recommendations to control resistant organisms, providing essential information in order to improve the quality of empiric antimicrobial prescribing or guiding development of antimicrobial policies. National and regional distributions of the data are important to enable local prescribing practices. The carbapenem (meropenem, imipenem)? with activity against *P. aeruginosa *have been the most active broad-spectrum antimicrobial class documented by numerous large surveillance programs [1]. One of the most cited global surveillance studies includes the Meropenem Yearly Susceptibility Test Information Collection (MYSTIC) program, an international resistance surveillance study which has been initiated 10 years ago with more than 100 participants worldwide [2-9]. The MYSTIC program is considered as a valuable tool to recognize the emergence of carbapenem and broad spectrum resistance mechanisms. An observatory surveillance system was established in 2006 to monitor the *in vitro *activity of meropenem following its approval in France in 2006 [10]. The major objective of the present study was to assess the *in vitro *activity of meropenem which has been recently re-introduced in French hospitals, compared to imipenem and piperacillin/tazobactam, against clinical isolates included in the spectrum of meropenem. One thousand and seventy one pathogens responsible for severe nosocomial infections were collected from 15 geographically diverse institutions in France. The secondary aim of the study was to compare the results of routinely testings and those obtained by a central laboratory.

The 15 French microbiology laboratories conducted susceptibility testing by disk diffusion test or broth dilution method according to the CA-SFM (Comité de l'Antibiogramme de la Société Française de Microbiologie) recommendations [11]. Since the participants of the 15 laboratories were using the CA-SFM breakpoints and because the EUCAST breakpoints will be introduced soon in France, approved CA-SFM and EUCAST (European Committee for Antimicrobial Susceptibility Testing [12]) interpretative breakpoints were used. The results obtained by the different laboratories (MICs by Etest method) were compared to those obtained by the centralized agar dilution method.

## Methods

Fifteen French Community and Universitary Hospital Centres participated in this study and were chosen to represent geographically separate areas. Clinical isolates were collected between January and June 2006.

### Isolates

The study protocol outlined specific quotas per medical centre among *E. coli *and other *Enterobacteriaceae*, non fermenter Gram-negative bacilli, *Staphylococci*, *Streptococcus pneumoniae *and anaerobes, for a total of 80 bacterial strains. Each centre collected the first 20 clinically relevant non-repetitive isolates of each selected species from patients hospitalized in 2006 with nosocomial infections: bacteremia, peritonitis, pulmonary infections and wound infections. Clinical and microbiological relevance was defined by "100 recommendations on nosocomial infections" from the French Health Ministry [13]. Species or genera known to be resistant to meropenem (methicillin-resistant -*Staphylococci*, *Enterococccus faecium *and *Stenotrophomonas maltophilia*) were excluded from the study collection.

### Antimicrobial susceptibility methods

Antibiotic susceptibility testing was carried out using the local centre routine methods (Vitek, Etest, disk diffusion method). Susceptibilities were determined for meropenem, imipenem and piperacillin/tazobactam (8/1). Oxacillin susceptibility of *Staphylococci *was determined by each individual centre using routine methodology. The detail of each method was provided by each participant. Etest method was used by each participant in order to determine MICs for the following agents: meropenem, imipenem and piperacillin/tazobactam (tazobatam at one concentration of 4 mg/L) according to the manufacturer's recommendations. All aerobic bacteria were tested onto Mueller Hinton agar. The medium was supplemented with 5% sheep blood for *S. pneumoniae *susceptibility testing. Susceptibilities of anaerobes were tested in a centralized laboratory. Meropenem, imipenem and piperacillin/tazobactam were tested by agar dilution method onto *Brucella *medium (DIFCO) according CLSI M11 A2 reference method and Etest method was realized on the carbapenems (meropenem, imipenem) only.

All bacteria were centralized and sent to a specific laboratory. Antimicrobial susceptibility testing was determined by Agar dilution reference method and disk diffusion tests using 30 μg disks (Biorad Laboratories, France) were performed for meropenem according to the CA-SFM recommendations [11]. The antimicrobials were obtained from the manufacturers: meropenem (AstraZeneca Pharmaceuticals, France) imipenem (Merck Laboratory, France) piperacillin/tazobactam (Wyeth Pharmaceuticals, France). The antimicrobials were tested at the following dilution range: meropenem and imipenem: from 0.016 to 256 mg/L; piperacillin/tazobactam: from 0.016/4 to 1,024/4 mg/L. MICs interpretative criteria followed published guidelines established by the CA-SFM (2006) or EUCAST (2006) where applicable. Quality controls including the following ATCC strains or CIP strains (Pasteur Institute Collection) were tested by each centre on the day of testing: *E. coli *ATCC 25922, *S. pneumoniae *CIP 104485, *P. aeruginosa *ATCC 27853, *Staphylococcus aureus *ATCC 25923.

### Beta lactamase screening

When suspected, Gram negative bacilli were screened for Expanded-Spectrum-Beta-Lactamase activity (ESBL) according to the "100 recommendations on nosocomial infections" from the French Health Ministry [13]; EBSL activity was confirmed by *in vitro *synergy between third generation cephalosporins and clavulanate (2 mg/L). Hyper or depressed production of AmpC beta-lactamase was identified by cloxacillin and by high level resistance to cephalosporins and piperacillin/tazobactam, with no change in susceptibility in the presence of clavulanate. All *P. aeruginosa *and *Acinetobacter *with an elevated carbapenem MIC result (≥ 2 mg/L) were screened for the presence of a metallo β-lactamase or another serine β-lactamase, using Etest beta-lactamase strip (AB Biodisk laboratories: Etest + EDTA and Etest + clavulanate) in presence or in absence of a concentration of cloxacillin in order to inhibit the presence of AmpC cephalosporinase.

### Ethical conduct of the study

The study has been conducted in accordance with "recommendations guilding physicians in biomedical research involving human patients " (declaration of Helsinki 1964). The study did not involved biological material or gentically modified organisms. All the strains involved in the study came from routine samples without any additionnal material.

### Statistical analysis

The statistical significance tests of differences between centres and between the two methods were carried out using analysis of variance and Student's t test. MICs versus zone diameter scattergram was prepared for meropenem. Very major error (VME) was defined as a difference superior of 2 log2 between the MICs carried out by the two methods, major error (ME) as a difference of 2 log2 and minor error (mE) as a difference of 1 log2.

## Results

A total of 1071 isolates were included in this study. They were isolated from bacteremia, (57%) peritonitis (11%), pneumonia (13%) or bronchitis (17%) and wound infections (2%).

### *In vitro *susceptibility results

Table 1 shows the ratio of susceptible strains to antibiotics, determined by the local study participants by routine methods: agar diffusion (56%), broth dilution (VITEK [36%] or ATB API [8%]). Table 1 also compares the rates of susceptible strains, according to the CA-SFM criteria, obtained for meropenem, imipenem and piperacillin/tazobactam, by the centralized agar dilution method and locally determined by Etest method.

**Table 1:** Susceptibility pattern of nosocomial infection agents obtained at local and central laboratories.

**Drugs**	**% Susceptibility (Number tested)**		
	**Locally determined**		**Centralized**
	
	**Routine**	**Etest**	**Agar DM**
			
*Escherichia coli*			
Meropenem	100 (92)	100 (139)	100 (139)
Imipenem	100 (139)	100 (139)	100 (139)
Piperacillin/tazobactam*	82 (139)	89 (139)	90 (139)
			
*Enterobacteriaceae (other than E coli)*			
Meropenem	100 (86)	99 (139)	99 (139)
Imipenem	99 (139)	97 (139)	97 (139)
Piperacillin/tazobactam*	78 (139)	86 (139)	99 (139)
			
*Pseudomonas aeruginosa*			
Meropenem	87 (87)	91 (129)	95 (129)
Imipenem	78 (137)	70 (129)	84 (129)
Piperacillin/tazobactam*	88 (136)	89 (129)	90 (129)
			
*Acinetobacter*			
Meropenem	88 (50)	87 (86)	95 (79)
Imipenem	94 (84)	94 (86)	97 (79)
Piperacillin/tazobactam*	79 (82)	80 (86)	100* (79)
			
*Other non fermentative bacteria*			
Meropenem	73 (33)	89 (33)	88 (33)
Imipenem	85 (41)	87 (33)	88 (33)
Piperacillin/tazobactam*	90 (41)	97 (33)	90 (33)
			
*Staphylococcus aureus (susceptible to methicillin)*			
Meropenem	-	100 (145)	100 (145)
Imipenem	-	100 (145)	100 (145)
Piperacillin/tazobactam*	-	100 (145)	100 (145)
			
*Coagulase negative staphylococci (susceptible to methicillin)*			
Meropenem	-	100 (129)	100 (129)
Imipenem	-	100 (129)	100 (129)
Piperacillin/tazobactam*	-	100 (129)	100 (129)
			
*Streptococcus pneumoniae*			
Meropenem	-	100 (136)	100 (136)
Imipenem	-	100 (136)	100 (136)
Piperacillin/tazobactam*	-	100 (136)	100 (136)

*Tazobactam: 4 mg/L

**Figure 1:**
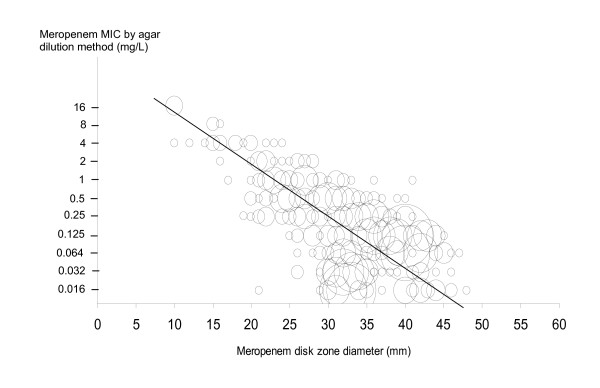
**Scattergram comparing meropenem disk zone diameters and log MICs by using agar dilution method for 725 strains (EUCAST values).** Minor errors figure in full circles.

Table 2 summarizes the MIC values inhibiting 50% (MIC50), 90% (MIC90) as well as the MIC range and the percentage of susceptible strains obtained by centralized agar dilution method according to EUCAST criteria. Figure 1 shows the concordance between disk zone diameter (mm) and MICs values for meropenem.

**Table 2:** Antimicrobial activity of meropenem, imipenem and piperacillin/tazobactam against 1071 pathogens determined by agar dilution reference method.

Species (number tested)	MICs (mg/L)			% Susceptiblity
Drugs				
	MIC50	MIC90	Range	EUCAST
*Escherichia coli (139)*				
Meropenem	0.016	0.016	0.016-0.032	100
Imipenem	0.064	0.125	0.032-0.5	100
Piperacillin/tazobactam*	2	8	0.25-128	90
				
*Enterobactericeae (other than E coli) (139)*				
Meropenem	0.032	0.064	0.016-16	99
Imipenem	0.125	1	0.032-16	98
Piperacillin/tazobactam*	2	16	0.125-128	90
				
*Pseudomonas aeruginosa (129)*				
Meropenem	0.5	4	0.064-16	84
Imipenem	2	16	0.5-32	84
Piperacillin/tazobactam*	8	32	0.25-256	90
				
*Acinetobacter (86)*				
Meropenem	0.5	2	0.125-16	94
Imipenem	0.5	1	0.125-8	98
Piperacillin/tazobactam*	0.032	2	0.032-16	100*
				
*Other non fermentative bacteria (30)*				
Meropenem	0.5	2	0.03-16	93
Imipenem	1	2	0.125-8	97
Piperacillin/tazobactam*	2	8	0.032-64	96
				
*Staphylococcus aureus (susceptible to methicillin) (145)*				
Meropenem	0.125	0.125	0.032-1	100
Imipenem	0.032	0.064	0.016-0.25	100
Piperacillin/tazobactam*	1	2	0.064-8	100
				
*Coagulase negative staphylococci (susceptible to methicillin) (129)*				
Meropenem	0.064	0.125	0.032-0.5	100
Imipenem	0.016	0.032	0.008-0.125	100
Piperacillin/tazobactam*	0.5	1	0.016-2	100
				
*Streptococcus pneumoniae (136)*				
Meropenem	0.016	0.25	0.016-0.25	100
Imipenem	0.016	0.125	0.016-0.25	100
Piperacillin/tazobactam*	0.25	4	0.016-8	100
				
*Anaerobes (138)*				
Meropenem	0.032	0.5	<0.016-4	100
Imipenem	0.064	0.5	<0.016-8	100
Piperacillin/tazobactam*	0.5	8	<0.016-32	100

*Tazobactam: 4 mg/L

#### Gram-negative pathogens

Susceptibility testing, determined by local participants, showed that carbapenems were the most effective drugs against *Enterobacteriaceae *and *Acinetobacter*, whereas piperacillin/tazobactam was the most effective drug against other non fermenter bacilli. The incidence of ESBL producers was 2% among *E coli*, 5% among other *Enterobacteriaceae *(2.5% among *Klebsiella pneumoniae*) and 0% among all non fermenter bacilli. No metallo beta-lactamase has been identified in *P. aeruginosa*.

Results obtained by agar dilution method demonstrated the lowest MICs values with meropenem for *Enterobacteriaceae*. MICs of meropenem were frequently 2-fold lower than MICs of imipenem for *Enterobacteriaceae *and *P. aeruginosa*. MICs observed for carbapenems in non-fermenter Gram-negative bacilli were higher. Following EUCAST breakpoints, no *E. coli *resistant to carbapenems were identified. One *Enterobacter cloacae *isolate was resistant both to imipenem and meropenem (MIC = 16 mg/L) and two *Proteus mirabilis *showed an intermediate susceptibility to imipenem (MICs = 4 mg/L); all other *Enterobacteriaceae *were fully susceptible to carbapenems. Following EUCAST criteria, the same proportion of susceptible strains was observed for meropenem and imipenem. Among non fermenter bacilli other than *P. aeruginosa*, one *Acinetobacter *and one *Alcaligenes *were resistant to meropenem (MICs: 16 mg/l) but respectively intermediate or susceptible (MIC respectively 8 mg/l and 2 mg/l) to imipenem. Figure 1 displays the scattergram of meropenem MICs values versus disc diffusion zone diameters. Error rate (1.6% of minor errors observed with Gram-negative bacilli) was within an acceptable range. Piperacillin/tazobactam demonstrated very good activity against Gram negative bacilli, particularly against non *P. aeruginosa*, aerobes, and bacilli. Discrepancies, which are the most important with that antibiotic, are related to the combination of piperacillin and tazobactam at various concentrations depending the method used.

#### Gram-positive pathogens

The *in vitro *activity of antimicrobials against Gram positive pathogens is displayed on Table 1. A total of 410 pathogens were tested. Table 2 gives the MIC50, the MIC90, the range and the percentage of susceptible strains. Against Gram-positive pathogens, imipenem frequently showed MICs one or two-fold lower than meropenem without any significant difference between the two compounds (p > 0.05). All strains were susceptible to imipenem, meropenem and piperacillin/tazobactam.

#### Anaerobes

**Figure 2:**
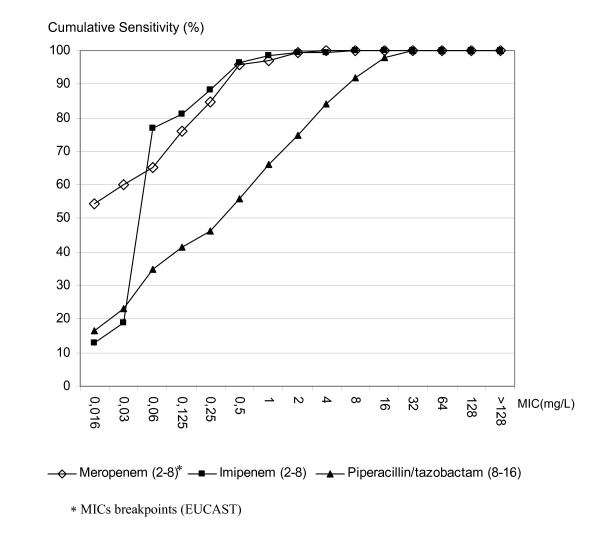
**Comparative activity of antibiotics against anaerobes. **Cumulative curves of MICs realized by agar dilution reference method.

Figure 2 shows the MIC cumulative curves determined by agar dilution method. The best activity was observed with meropenem and imipenem (MIC90 = 0.5 mg/L), followed by imipenem (MIC90 = 8 mg/L).

### Method comparison for Carbapenem MICs results

Comparison between agar dilution method and Etest method shows correlations of 0.72 for meropenem and 0.74 for imipenem. Table 3 illustrates that 40% (n = 367) of the results were identical for meropenem and 23% (210) for imipenem. Respectively, for meropenem and imipenem, 81.4% and 61% of the strains tested were within +/- 1 log2 (mE) dilution and 96.6% and 91.5% within +/- 2log2 (ME) dilution. A significant trend toward a higher Etest MIC result was observed among these results for the two compounds (p < 0.01). The trend toward a higher Etest MIC was higher for imipenem (1.1 log2 dilution) than for meropenem (0.5 log2 dilution).

**Table 3:** Variation of meropenem MIC and imipenem MIC results obtained by Etest method realized by local participants, and by agar dilution reference method realized by a centralized centre (anaerobes excluded).

	% Variations from Agar Dilution Method MIC in log2 dilutions							
	
Etest	VME >-2	ME -2	mE -1	Same	mE +1	ME +2	VME >+2	Log trend *
Meropenem n = 923	7	9	78	367	306	132	24	+0.5
(%)	(0.8)	(2.2)	(8.5)	(40)	(33)	(14)	(2.5)	

Imipenem n = 923	5	12	37	210	312	273	74	+1.1
(%)	(0.5)	(1.3)	(4)	(22.7)	(33.5)	(29.7)	(8)	

* Trend in MIC when compared Etest/Agar dilution method

VME: very major error - ME: major error - mE: minor error

#### Factors influencing inter method categorical MIC errors

Three percent of VME (31 strains) were observed for meropenem. Gram-negative bacilli were over represented with 27/31 strains causing VME. All bacterial groups were represented. We did not identify any influence of a centre effect. Among the 15 centres, 0 to 6 VME per centre were observed. The range of agreement among the 15 centres was 68% to 98% (within +/- 1log).

Seventy nine ME (8.5%) were observed with imipenem. Gram-negative bacilli (GNB) or Gram-positive cocci (GPC) were involved (8% of the GNB and 7% of the GPC). A significant centre effect was observed (p < 0.01): 57% of ME observed with GPC were due to 2 centres. Quality controls realized by each participant during the study allowed the identification of errors by the two local participants. Among the 15 centres, 10 had 0% to 5% of ME, 3 had 7 to 16% of ME and had not been identified by the quality control. Two had more than 30% of ME.

## Discusion and Conclusions

This study was the first national prospective surveillance study assessing antimicrobial activity of meropenem against recent clinical isolates from French hospitals. Antimicrobial susceptibility surveillance studies play a fundamental role in the fight to control resistant organisms. Harmonization between methods and interpretations are important to compare the results of various surveys located in different geographic areas [14,15]. The EUCAST group has provided progress toward harmonization of susceptibility testing methods used in European Nations and an international consensus on breakpoint definitions. Since 2008, the CA-SFM recommends the EUCAST methods and breakpoints. In this study, we report that meropenem, imipenem and piperacillin/tazobactam are very active against Gram-negative bacilli, including *Enterobacteriaceae*, *P. aeruginosa*, *Acinetobacter *and other non fermenter-bacilli. The susceptibility data obtained from this multicentre study were similar to data previously published for studies conducted in Canada and other European countries [9,16]. Over the last 5 years, *E coli *susceptibility worldwide has shown a trend to decrease, in particular to fluoroquinolones and beta-lactams, in relation with the dissemination of CTX-M producers and/or of an increase of AmpC [16-20]. Meropenem and imipenem demonstrate good activity against *Enterobacteriaceae*, including strains producing ESBLs or AmpC (100% for *E coli*, 99% for other *Enterobacteriaceae*), meropenem usually being 2 to 4 fold more potent than imipenem [21-23]. Susceptibility of *Acinetobacter *was close to 94 to 98% which is similar to other studies reporting susceptibility in Europe and the USA [8,16]. In this report, piperacillin/tazobactam was the most potent antibiotic against *P.aeruginosa *(90% of susceptible strains versus 84% for carbapenems) as reported in other studies [5]. Carbapenems as piperacillin/tazobactam showed a very good activity with low MICs against Gram-positive pathogens (MSSCN, MSSA and *S. pneumoniae*).

Communication of national and regional surveillance data are important to enable local prescribing practices, but in reality, every day antibiotic therapy is based on the sensitivity of antibiotic produced locally. Numerous studies have demonstrated the validity of the Etest method compared to the agar dilution method. The variability of inter-laboratory results is always tested by using reference strains but the statistical analysis of the centre results compared the global data is able to give rise to important informations, it reflects the reality of the daily work. The rate of agreement varies relatively to the antibiotic tested or the bacterial species [24-26]. The agreement (including minor errors) observed between the 2 testing methods was >80% for meropenem, but was low for imipenem (61%). The VME for meropenem were observed in Gram-negative bacilli, whereas there was no difference between Gram-negative bacilli and Gram-positive cocci for imipenem. In general, MIC values obtained using the Etest method were commonly higher than values obtained using agar dilution method. On occasion, interpretation of the MIC could have significant consequences on result reporting (S, I, R) to the physician and subsequent prescribing. Analysis of the VME showed that no particular species was concerned but 2 centres presented VME (p < 0.05). This observation suggested us that it could be due to a local technical problem such as inapropriate storage. Carbapenems and particularly imipenem are unstable antibiotics. The humidity could alter the quality of the strips and could modify the results.

## Competing interests

The authors declare that they have no competing interests.

## Authors' contributions

MLJG participated in the conception, study design, statistical analysis, coordination, supervision of laboratory analysis and manuscript writing. MK, JDC, MC, LD, JM, CMS and MRD participated in study design and supervision of laboratory testing. All authors read and approved the final manuscript

## Pre-publication history

The pre-publication history for this paper can be accessed here:

http://www.biomedcentral.com/1471-2334/10/72/prepub
